# Vital Statistics of Texas Bulletin for November, 1907

**Published:** 1908-01

**Authors:** W. M. Brumby

**Affiliations:** State Health Officer of Texas


### Abstracts and Selections.

### Vital Statistics of Texas Bulletin for November, 1907.

Our system of Vital Statistics is in embryo, but it is growing fast and, with proper encouragement and support, can and must be made a success. Since it is comparatively new to the people, it will require a considerable amount of education for them to be made to realize its value, and knowing that the press is the most powerful and efficient means we have of bringing matters of public interest before the people and that they also, realizing the exigencies of the case, are in a position to enlighten the people, I appeal to them for their co-operation in furthering the cause of vital statistics, and to assist us in molding public sentiment in order that physicians, midwives and others be forced to make more accurate reports and our next Legislature more ample appropriation.

The law provides that all births and deaths must be reported promptly to the county clerk by physicians, surgeons, midwives and accoucheurs in attendance of the same under penalty of from $5 to $50, and that the county clerk under similar penalty keep the same as a permanent record and supply a copy to the State Health Officer, monthly. Every effort has been made to get those interested to make proper reports to this office, and a large number of physicians are complying with the law, but there are still a large number that fail to report, and while I desire to maintain amicable relations between the members of the profession and this department, I shall insist on the enforcement of the law, and expect to make an example of some of the profession at an early date. While we have recourse to law, yet laws not backed by public sentiment are seldom of any consequence; therefore, I am anxious that the public be impressed with the necessity for this work, and urge them to give their co-operation by insisting that all physicians, midwives and others make prompt and accurate report.

---

It would seem that there were but few people whose birth, marriage or death has not or will not, at some time, become a matter of legal cognizance, and hardly a relation in life in which such a record may not be evidence of greatest value.

"The correct registration of its birth is a valuable inheritance to which every child is entitled and which is within the power of every parent to bestow."

For the month of October, 801 deaths were reported, and for November, 1067, showing an increase of 34 per cent; also, 11 per cent increase in births is noted, which is encouraging, but should and I hope will be improved upon each month until accurate returns are to be had.

Physicians in some of the cities are objecting to our birth and death certificate blanks. We should like to call their attention to the fact that every registration State is using this form, and that it is the form adopted by the Census Bureau of Washington and approved by the American Public Health Association, and is complete in every detail. Every blank and question asked is essential; in fact, indispensable.

BIRTHS.

| | |
|---|---|
| Males | 2529 |
| Females | 2217 |
| White | 4441 |
| Black | 305 |
| Alive | 4621 |
| Stillborn | 125 |
| Total | 4746 |

DEATHS CLASSIFIED BY AGES.

| | |
|---|---|
| Under 1 year | 204 |
| From 1 to 5 | 64 |
| From 5 to 10 | 36 |
| From 10 to 20 | 59 |
| From 20 to 30 | 123 |
| From 30 to 40 | 119 |
| From 40 to 50 | 67 |
| From 50 to 60 | 71 |
| Over 60 | 212 |
| Not given | 119 |
| Totals for all ages | 1074 |

W. M. BRUMBY,
State Health Officer of Texas.

